# Prenatal Emotion Dysregulation, Respiratory Sinus Arrhythmia, and Mindfulness Predict Toddler Socioemotional Development

**DOI:** 10.1007/s10802-026-01454-x

**Published:** 2026-04-29

**Authors:** Anna M. Compton, Sarah E. Maylott, Anna M. Zhou, Kira Wright, Nicolette C. Molina, Madeleine Bruce, Alec Westover, K. Lee Raby, Sheila Crowell, Elisabeth Conradt

**Affiliations:** 1 Department of Psychology, Wayne State University, Detroit, MI USA; 2 Department of Psychiatry and Behavioral Sciences, Duke University, Durham, NC USA; 3 Department of Psychology, The University of Utah, Salt Lake City, UT USA; 4 Department of Psychiatry, The University of Colorado Anschutz Medical Campus, Denver, CO USA; 5 Department of Psychology, The University of Denver, Denver, CO USA; 6 Department of Psychology, The University of South Carolina, Columbia, SC USA; 7 Department of Psychology, The University of Oregon, Eugene, OR USA

**Keywords:** Prenatal emotion dysregulation, Mindfulness, Social emotional development, Infancy, Toddlerhood

## Abstract

Socioemotional skills are essential for healthy development, shaping emotional health, interpersonal functioning, and long-term academic success. Disruptions in socioemotional development can emerge as early as infancy and may have lasting consequences. This study examined how prenatal maternal emotion dysregulation, assessed via self-report and respiratory sinus arrhythmia (RSA), related to toddler socioemotional outcomes at 18 months, and whether maternal mindfulness, measured via everyday mindful attention and awareness, moderated these associations. Participants were 385 mother–toddler dyads from a longitudinal study on maternal emotion dysregulation. Toddler socioemotional outcomes were assessed at 18 months through parent report. We tested associations between maternal emotion dysregulation and toddler outcomes, with mindfulness examined as a moderator. Findings suggest that higher self-reported maternal emotion dysregulation is associated with toddler socioemotional difficulties (i.e., internalizing, externalizing, and dysregulation), while higher prenatal baseline RSA predicts greater toddler competence. Additionally, maternal mindfulness moderated these associations, amplifying the positive association between prenatal RSA and toddler competence and the negative association between self-reported prenatal emotion dysregulation and competence. These findings underscore the critical role of maternal well-being during pregnancy and highlight that prenatal mindful attention and awareness plays a role in toddler socioemotional development.

**Supplementary Information:**

The online version contains supplementary material available at 10.1007/s10802-026-01454-x.

Socioemotional skills develop early and lay the foundation for emotional health, relationships, mental health, and academic success (Alzahrani et al., 2019; Goodman et al., 2015). These skills include the capacity to engage with others, manage frustration, express a range of emotions, and adapt to changes, and are assessed through competencies (e.g., compliance, attention, empathy) alongside emerging problems in areas such as externalizing, internalizing, and dysregulation (Carter et al., 2003). Disruptions in infant behavioral and emotional development have been linked to higher risk for internalizing and externalizing psychopathology, greater symptom severity, and increased mental health service use (Bagner et al., 2012; Briggs-Gowan et al., 2006; Essex et al., 2009). Although socioemotional difficulties can emerge in toddlerhood and remain stable across childhood (Briggs-Gowan et al., 2006; Essex et al., 2009), little is known about early experiences that shape socioemotional functioning during this period of plasticity. Clarifying these early influences is key to identifying at-risk infants and guiding preventive interventions.

The Developmental Origins of Health and Disease (DOHaD) theory posits that in-utero environmental conditions can shape the physiological and metabolic pathways of the developing fetus, influencing health and disease susceptibility across the lifespan (Barker, 1998, 2007; Gluckman et al., 2016). Studies have shown that children of mothers with prenatal depression or anxiety are at higher risk for psychopathology diagnoses later in development (Monk et al., 2019). However, less work has examined how maternal transdiagnostic factors —processes that cut across traditional diagnostic categories and confer risk for multiple forms of psychopathology—such as emotion dysregulation, may relate to the development of socioemotional problems and/or competencies during infancy and toddlerhood. This is especially important as the field moves toward transdiagnostic and dimensional approaches to diagnosis (Kotov et al., 2017). Furthermore, few studies have explored whether maternal mindfulness during pregnancy, can offset children’s risk for adverse socioemotional outcomes. The current study aims to address these gaps in the literature by examining: (1) the longitudinal relations between prenatal maternal emotion dysregulation and toddler socioemotional development and (2) whether mindfulness serves as a protective factor in these associations.

## Intergenerational Implications of Prenatal Emotion Dysregulation

Emotion dysregulation refers to difficulties managing emotional experiences and responses (Crowell et al., 2020; Gratz & Roemer, 2004), such as identifying emotions or reacting with disproportionate intensity or duration (Thompson, 2019). It is a transdiagnostic marker of psychopathology, linked to both internalizing and externalizing disorders (Beauchaine & Cicchetti, 2019; Sloan et al., 2017). Emotion dysregulation disrupts goal-directed behavior and impairs functioning across relationships, health, and daily life. It has been associated with depression, anxiety (Ehring et al., 2010), self-harm (Gratz & Roemer, 2004), and anxious attachment (Cronin et al., 2018). Given its broad impact, understanding how fetal exposure to maternal emotion dysregulation shapes development is essential.

Emotion dysregulation is a multilevel construct that can be assessed through physiological indices of regulatory capacity, such as parasympathetic nervous system activity (e.g., RSA), as well as through self-report measures that reflect individuals’ subjective awareness and appraisal of their emotional experiences (Beauchaine, 2015). Respiratory sinus arrhythmia (RSA), a measure of heart rate variability associated with the respiratory cycle, is considered a reliable biomarker of emotion dysregulation (Beauchaine, 2015). Baseline RSA reflects one's capacity to respond adaptively to environmental challenges, and individuals exhibiting higher baseline RSA often display more efficient modulation of cardiac activity and emotional arousal (Beauchaine, 2001, 2015; Porges, 2007). Conversely, lower baseline RSA is characterized by less parasympathetic engagement during rest and has been linked to heightened vulnerability to psychopathology and poor health (Beauchaine, 2001; Yaptangco et al., 2015). Indeed, pregnant individuals with lower baseline RSA were more likely to report depression and self-injurious thoughts (Lin et al., 2019), and poor maternal mental health during the perinatal period predicts socioemotional difficulties in early childhood (Duguay et al., 2022; O’connor et al., 2002; Porter et al., 2019). These findings suggest that lower prenatal RSA may be linked to infant socioemotional problems. In sum, emotion dysregulation during pregnancy may represent one pathway of intergenerational mental health.

A small but growing body of literature supports this hypothesis that prenatal maternal emotion dysregulation—assessed via self-report or baseline RSA—predicts infant social and emotional outcomes. For instance, using the same longitudinal cohort as the present study, higher levels of self-reported prenatal maternal emotion dysregulation were associated with neurobehavioral indicators of infant emotion dysregulation at birth, including inattention and blunted arousal (Ostlund et al., 2019). In a follow-up analysis with the same sample, greater prenatal maternal emotion dysregulation was indirectly associated with toddlers’ expressive vocabulary at 18 months, through increased postpartum perceived everyday stress (Wright et al., 2025). In addition, lower prenatal maternal resting RSA directly predicted smaller toddler expressive vocabulary. In a similar line of research, maternal and paternal psychological inflexibility during pregnancy—a component of emotion dysregulation—and their associations with toddler socioemotional difficulties were examined in the context of mindful parenting (Laifer et al., 2025). However, their study did not include RSA, which is important for identifying early physiological risk indicators for later socioemotional difficulties.

### Protective Effects of Maternal Mindfulness

There are protective factors during the prenatal period that may modulate and buffer the effects of maternal emotion dysregulation—a transdiagnostic feature of psychopathology—on infant outcomes. One such factor is maternal mindfulness, which is defined as “paying attention in a particular way: on purpose, in the present moment, and nonjudgmentally” (Kabat-Zinn, 2011). Mindfulness helps individuals develop a curious and objective attitude towards both positive and negative experiences, which has been shown to lessen avoidant and reactive behaviors to strong affective stimuli (Bishop et al., 2004). Mindfulness is a trainable skill that has been positively associated with numerous aspects of psychological health, including increased life satisfaction, empathy, and emotional health (Keng et al., 2011).

Maternal mindfulness may help support pregnant women's emotional well-being. For example, higher self-reported mindfulness scores have been associated with lower levels of emotion dysregulation, emotional distress, and maternal anxiety in pregnant individuals (Braeken et al., 2017; Ostlund et al., 2019; Van den Bergh et al., 2005). A randomized controlled trial found that a 6-week mindfulness intervention significantly reduced pregnancy-specific and related anxiety compared to a reading control group (Guardino et al., 2014). Prenatal mindfulness has also been associated with improved infant outcomes, including better adaptive functioning, stronger neural responses, and fewer self-regulation and negative affectivity problems (Braeken et al., 2017; Van den Bergh et al., 2005). In one study, higher maternal emotion dysregulation predicted lower newborn attention, but only among mothers with low mindfulness (Ostlund et al., 2019). These findings provide preliminary support for the idea that maternal mindfulness may buffer against some negative effects of prenatal exposure to maternal emotion dysregulation and promote toddler socioemotional functioning. However, few studies have examined whether mindfulness moderates the link between maternal emotion dysregulation and toddler socioemotional functioning.

### Current Study

The overarching goal of the present study is to examine the role of prenatal emotion dysregulation (measured via self-report and peripheral nervous system markers of maternal baseline RSA) on toddler socioemotional development at 18-months. Further we examine prenatal maternal mindfulness, assessed as everyday mindful attention and awareness, as a potential moderator of the associations between prenatal emotion dysregulation and toddler socioemotional outcomes. The first aim of this study was to examine the main effects of maternal prenatal emotion dysregulation and maternal prenatal resting RSA on toddler externalizing, internalizing, dysregulation, and competence scores at 18 months. We expected that higher self-reported emotion dysregulation (Aim 1a) and lower resting RSA (Aim 1b) would predict higher toddler externalizing, internalizing, and dysregulation, and lower competence at 18 months.

The second aim was to test whether prenatal maternal mindfulness moderates relations between prenatal emotion dysregulation and the same toddler socioemotional outcomes. We expected that higher self-reported emotion dysregulation and lower resting RSA would predict more socioemotional difficulties and lower competence in infants, but that these effects would depend on maternal mindfulness scores. Specifically, we hypothesized that maternal mindfulness may have a buffering effect such that prenatal risk factors would no longer be significantly associated with socioemotional difficulties. In contrast, at lower levels of mindfulness, we hypothesized that higher self-reported emotion dysregulation and lower resting RSA would be more strongly linked to socioemotional difficulties. This pattern would suggest that mindfulness may serve as a protective factor against early developmental risk. All hypotheses and analytic approaches were pre-registered and can be accessed on the Open Science Framework (https://osf.io/7rsxc/overview?view_only=df79e0ac87264d18b26375989d36cb32).

## Method

### Participants

Participants include 385 mother-infant dyads from a larger longitudinal study examining the intergenerational transmission of emotion dysregulation from the third trimester of pregnancy to 36-months postpartum. Participants were recruited from local obstetric clinics in Salt Lake City, Utah and via email. Interested participants completed the Difficulties in Emotion Regulation Scale (DERS; Gratz & Roemer, 2004) and reported eligibility criteria items during screening. Eligible pregnant participants were ages 18–40, had no pregnancy complications (e.g., gestational diabetes or preeclampsia), no illicit substance use, and anticipated a singleton delivery. Participants with high and low self-reported emotion dysregulation scores were intentionally oversampled to achieve a more uniform distribution of emotion dysregulation. This was accomplished by dividing emotion dysregulation scores into bins: low 36–67, medium 68–89, and high 90–180; participants were determined ineligible if they fell into a bin that was already full. Supplemental Fig. 1 contains detailed information about participant recruitment and enrollment. Demographic data were collected during the prenatal timepoint (see Table 1). Overall, the sample reflected a broad range of psychosocial risk, characterized by diverse income levels and elevated prenatal maternal emotion dysregulation.

**Fig. 1:**
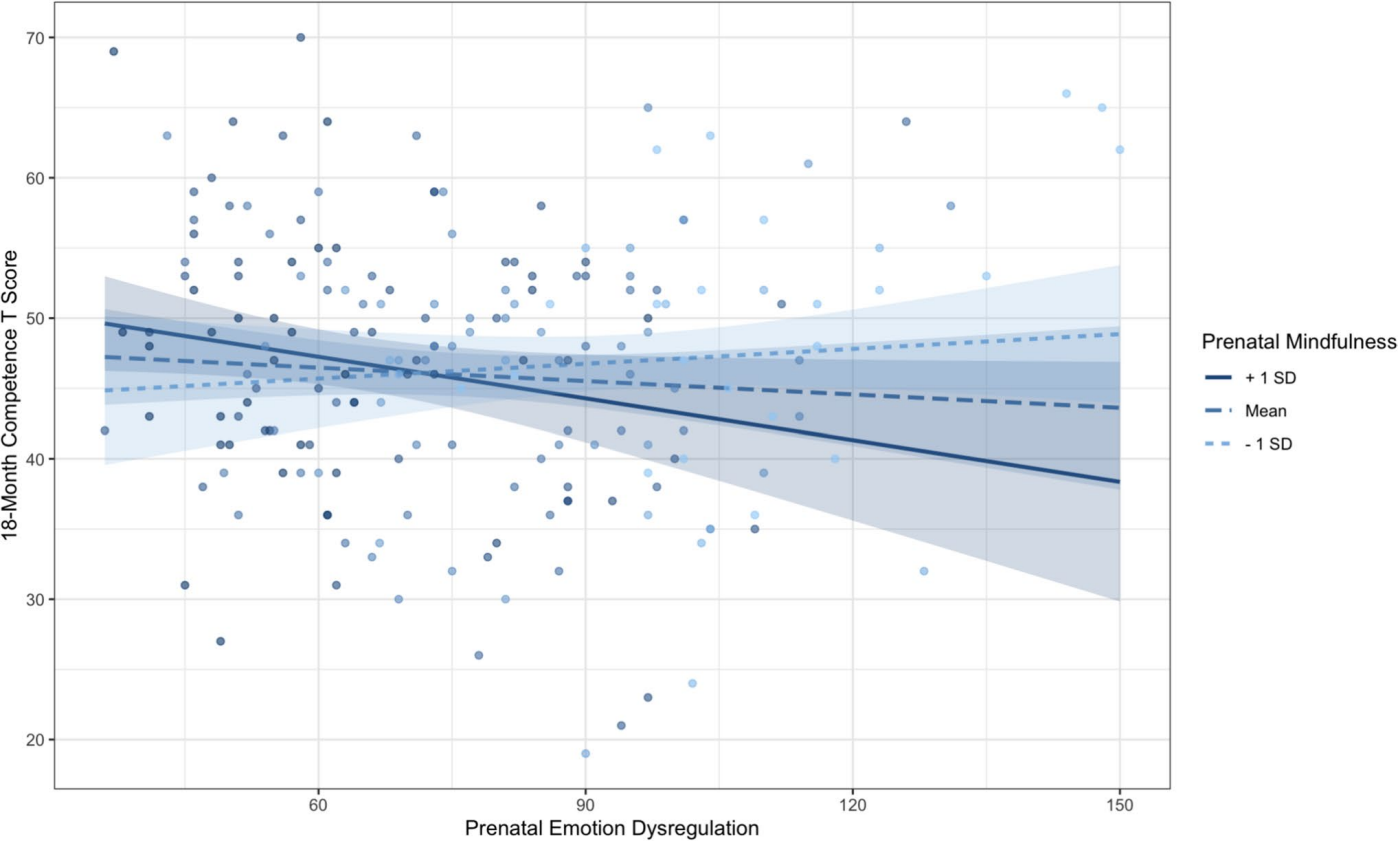
Interaction between prenatal self-reported maternal emotion dysregulation and prenatal maternal mindfulness predicting toddler competence at 18-months with 95% confidence intervals. The simple slope for high prenatal mindfulness (+ 1 *SD*; solid line) was significant (*p* < .05), while the slopes for mean and low mindfulness were not significant

**Table 1:** Participant demographics

Demographic Variable	N	Percent	Mean (SD)	Min–Max
Maternal Age in Years (at prenatal visit)	384	99.7%	29.35 (4.85)	18–40
Maternal Race and Ethnicity	384	99.7%		
American Indian or Alaskan Native and not Hispanic/Latina	5	1.3%		
American Indian or Alaskan Native and Hispanic/Latina	2	0.5%		
Asian and not Hispanic/Latina	26	6.8%		
Native Hawaiian or Other Pacific Islander and not Hispanic/Latina	7	1.8%		
Black or African American and not Hispanic/Latina	10	2.6%		
Black or African American and Hispanic/Latina	1	0.3%		
White and not Hispanic/Latina	219	56.9%		
White and Hispanic/Latina	65	16.9%		
Multiracial and not Hispanic/Latina	13	3.4%		
Multiracial and Hispanic/Latina	3	0.8%		
Prefer to self-report	12	3.1%		
Self-report race and ethnicity as Hispanic/Latina	7	1.8%		
Decline to answer	14	3.6%		
Maternal Education	381	99.0%		
Less than 12th grade	13	3.4%		
High school graduate or equivalent	45	11.7%		
Some college or technical school	114	29.6%		
College graduate	122	31.7%		
Any graduate school	87	22.6%		
Household Income	374	97.1%		
Under $9,000	20	5.2%		
$9,000—$14,000	12	3.1%		
$15,000—$19,999	10	2.6%		
$20,000—$24,999	10	2.6%		
$25,000—$29,999	17	4.4%		
$30,000—$39,999	32	8.3%		
$40,000—$49,999	24	6.2%		
$50,000—$79,999	112	29.1%		
$80,000—$99,999	48	12.5%		
$100,000 or more	89	23.1%		
Maternal Relationship Status	382	99.2%		
Married	310	80.5%		
Not married	59	15.3%		
Separated or divorced	12	3.1%		
Prefer to self-report	1	0.3%		
Infant Birth Order	313	81.3%	2.04 (1.06)	1—6
Infant Sex	381	99.0%		
Female	199	51.7%		
Male	182	47.3%		
Infant Gestational Age in Days (at delivery)	380	98.7%	274 (8.04)	239—294
Infant Birth Weight in Grams	375	97.4%	3329 (441.7)	1750–4560

## Measures

### DERS

The DERS (Gratz & Roemer, 2004) was assessed during the third trimester of pregnancy and is a 36-item self-report measure used to assess emotion dysregulation. The DERS consists of six subscales to capture emotion dysregulation: nonacceptance of emotional responses, difficulty engaging in goal directed behavior, impulse control difficulties, lack of emotional awareness, limited access to emotion regulation strategies, and lack of emotional clarity. The scale is administered on a 5-item Likert scale (*1—almost never* and *5—almost always*), has 11 reverse scored items, and consists of items such as “I pay attention to how I feel” and “When I’m upset, my emotions feel overwhelming.” A total score is computed by summing all items, with higher scores representing greater emotion dysregulation. The DERS has demonstrated good internal consistency for the study sample (Cronbach’s alpha = .96).

### Maternal Baseline RSA

Maternal electrocardiogram (ECG) data were collected continuously during the third trimester prenatal lab visit using a three-lead configuration and processed using MindWare (MindWare Technologies Ltd.; version 3.1.5). During the prenatal visit, expectant mothers contributed physiological data using heart rate monitoring equipment that was set up by trained research assistants in the lab (*n* = 58; 26%) or via Zoom (*n* = 168; 74%), with staff guiding the participant remotely (see Gao et al., 2021), for details on remote data collection during the COVID-19 pandemic). ECG data were processed in 60-s epochs by trained personnel, and RSA, the high-frequency band of the power spectrum waveform, was then calculated. Given higher respiration rates during pregnancy, we computed RSA using the high frequency band of .24-.60 Hz in line with recommendations to adjust frequency bands based on respiration rates (Shader et al., 2018). Resting RSA was calculated as the mean across all 10 epochs of the baseline task. Most participants with usable baseline data contributed all 10 epochs; however, 17 participants had missing data for one or more epochs (*M* = 3.05, Range = 1–8 epochs). After the data were initially cleaned, they were double-checked by the primary investigator to see if RSA values fell outside the expected range of 2–10. Importantly, RSA did not statistically differ between the lab (*M* = 5.63, *SD* = 1.11) and home visits (*M* = 5.56, *SD* = 1.39;* t*(116) = 0.41, *p* = .683.

### Mindful Attention and Awareness Scale (MAAS)

The Mindful Attention and Awareness Scale (MAAS; Brown & Ryan, 2003) was administered in the third trimester of pregnancy and consists of 15-items assessing the role of everyday mindful attention and awareness; that is, present-moment attention to and awareness of ongoing experiences. The MAAS primarily captures the attentional component of mindfulness and does not directly assess other facets often emphasized in broader conceptualizations of mindfulness, such as nonjudgemental acceptance, nor does it assess engagement in mindfulness-based practices or interventions. The MAAS uses a 6-point Likert scale (*1-almost always* and *6-almost never*) and includes items such as “I could be experiencing some emotion and not be conscious of it until sometime later.” For a total score, the mean is computed from the 15-items with higher scores indicating higher levels of mindfulness. The MAAS demonstrated good internal consistency for the sample (Cronbach's alpha = .91).

### Infant and Toddler Social and Emotional Assessment (ITSEA)

The Infant and Toddler Social and Emotional Assessment (ITSEA; Carter et al., 2003) was administered to parents when toddlers were 18 months old and consists of 166 items that measure socioemotional development in children ages 12–35 months. The ITSEA contains four domains to capture socioemotional development: externalizing (e.g., defiance, impulsivity, aggression), internalizing (e.g., withdrawal, fearfulness, sadness), dysregulation (e.g., difficulties with attention, sleep, and eating), and competence (e.g., compliance, empathy, attention, and prosocial behaviors; see Supplemental Table 1 for example items). The measure is administered on a 3-point Likert scale (*0-Not true* and *3-Very true*) and has 7-items that are reverse-scored. The ITSEA has psychometric support for test–retest and interrater reliability, as well as strong construct validity (Gleason et al., 2011). In the current sample, the ITSEA demonstrated adequate internal consistency across domains, with Cronbach’s alpha values of .83 for externalizing, .78 for internalizing, .83 for dysregulation, and .87 for competence.

### Procedure

Upon consenting to participate in the longitudinal study but prior to the third trimester prenatal lab visit, eligible participants completed online self-report measures of emotion dysregulation, mindfulness, and demographics prior to their third trimester lab visit. Physiological data were then collected during the visit, either in person or remotely via Zoom with staff guidance (For remote data collection procedures during COVID-19 see Gao et al., 2021). Resting RSA was collected continuously over a 10-min period in which the participant was instructed to relax and not engage in any other tasks. Participants were contacted and re-consented before participating in the 18-month lab or remote research visit and were asked to complete the ITSEA via an online link prior to the visit. Participants received up to $130 in compensation for completing the prenatal study visit and questionnaires, along with 18-month questionnaires. All study procedures involving human subjects were approved by the Institutional Review Board at the University of Utah.

### Analytic Plan

A series of longitudinal path models were estimated using the *lavaan* package (Rosseel, 2012) in R software platform. First, we ran two models to test whether prenatal self-reported emotion dysregulation (model 1; Aim 1a) and maternal prenatal resting RSA (model 2; Aim 1b) were associated with toddler socioemotional development at 18-months (i.e., externalizing behaviors, internalizing behaviors, dysregulation, and competence). Next, we ran two additional path models to examine whether prenatal maternal mindfulness moderated the associations between prenatal self-reported emotion dysregulation (model 3; Aim 2a) or maternal resting RSA (model 4; Aim 2b) and toddler socioemotional development. Interaction terms were grand mean centered to reduce the risk of nonessential multicollinearity. We then probed significant interactions by calculating the simple slope of maternal emotion dysregulation at three levels of maternal mindfulness, which correspond to average (mean), low (mean –1 *SD*), and high (mean + 1 *SD*) mindfulness levels. Additionally, regions of significance were examined using the Johnson–Neyman technique (Johnson & Neyman, 1936) to identify the range of maternal mindfulness values for which the association between maternal emotion dysregulation and the outcome was statistically significant.

### Missing Data

During the third trimester of pregnancy, 383 participants completed the Difficulty in Emotion Regulation Scale (DERS), 374 completed the mindfulness questionnaire, and 360 contributed usable resting RSA data. At 18 months postpartum, 261 participants (68%) completed a questionnaire regarding their toddler’s socioemotional development. Mothers who did and did not complete the 18-month questionnaires did not differ significantly with respect to maternal age, gestational age at birth, maternal parity, household income, infant sex, or prenatal emotion dysregulation. The only significant attrition difference was maternal education, such that mothers who possessed a four-year college degree at the prenatal timepoint were more likely to complete the 18-month questionnaires (χ^2^ = 10.74, *p* = .03). We tested maternal education in the models and all results remained. Further, maternal education was not significantly associated with mindfulness *r*(369) = − 0.03, *p* = .591. Although parent education may play a role in parenting behavior, in the present sample it showed no meaningful relation to the constructs of interest and therefore was not included in subsequent analyses, consistent with recommendations to avoid unnecessary adjustments (Wysocki et al., 2022). Missing data were handled using full information maximum likelihood for the full sample (*N* = 385; Allison, 2003), consistent with methodological guidelines for handling missing data (Little et al., 2024).

## Results

### Descriptive Statistics

Higher prenatal emotion dysregulation was related to lower mindfulness. Higher prenatal emotion dysregulation was also related to greater toddler internalizing, externalizing, and dysregulation behaviors. Higher maternal prenatal resting RSA was related to greater toddler competence. Lastly, higher prenatal maternal mindfulness was related to lower toddler dysregulation, and externalizing behavior. See Table 2 for full results.

**Table 2:** Descriptive statistics and correlations with confidence intervals

	*M* (*SD*)	*N*	**1**	**2**	**3**	**4**	**5**	**6**	**7**
*Prenatal Variables*									
1. Maternal DERS	80.11 (25.29)	383	_						
2. Maternal baseline RSA	5.45 (1.19)	360	-.07 [-.17, .04]	_					
3. Maternal MAAS	4.24 (0.86)	374	-.59** [-.66, -.52]	.06 [-.04, .17]	_				
*18-Month Variables*									
4. Toddler Externalizing Behavior	48.31 (7.87)	207	.18* [.04, .30]	-.08 [-.21, .07]	-.18* [.31, -.04]	_			
5. Toddler Internalizing Behavior	49.29 (10.73)	257	.13* [.01, .25]	-.01 [.14, .11]	-.09 [-.21, .03]	.27** [.13, .39]	_		
6. Toddler Dysregulation	43.55 (12.32)	260	.17** [.05, .29]	-.09 [-.21, .04]	-.15* [-.27, -.03]	.44** [.32, .54]	.47** [.37, .56]	_	
7. Toddler Competence	47.01 (9.50)	199	.00 [-.14, .14]	.17* [.02, .30]	-.04 [-.18, .10]	-.03 [-.17, .11]	-.08 [-.22, .06]	-.26** [-.39, -.13]	_

*Note.* * *p* < .05; ** *p* < .01. DERS = Difficulties in Emotion Regulation Scale; RSA = Respiratory sinus arrhythmia; MAAS = Mindful Attention and Awareness Scale; SD = Standard deviation. Values in square brackets indicate the 95% confidence interval (CI) for each correlation coefficient

### Prenatal Emotion Dysregulation, Resting RSA, and Toddler Socioemotional Outcomes

We first tested main effects of prenatal emotion dysregulation and resting RSA on toddler socioemotional outcomes. Path models revealed positive associations between prenatal emotion dysregulation and toddler externalizing (β = 0.20, *p* = .003, 95% CI [0.07, 0.34]), internalizing (β = 0.13, *p* = .029, 95% CI [0.01, 0.25]), and dysregulation (β = 0.18, *p* = .004, 95% CI [0.06, 0.29]). However, there were no significant associations between prenatal emotion dysregulation and competence, (β = −0.01, *p* = .87, 95% CI [−0.16, 0.13]; Supplemental Fig. 2). In contrast, prenatal maternal resting RSA was positively associated with toddler competence (β = 0.16, *p* = .024, 95% CI [0.02, 0.30]), but not externalizing (β = −0.08, *p* = .27, 95% CI [−0.21, 0.06]), internalizing (β = −0.01, *p* = .92, 95% CI [−0.13, 0.12]), or dysregulation (β = −0.09, *p* = .17, 95% CI [−0.21, 0.04]; Supplemental 3). See Supplemental Table 2 for full results.

**Fig. 2:**
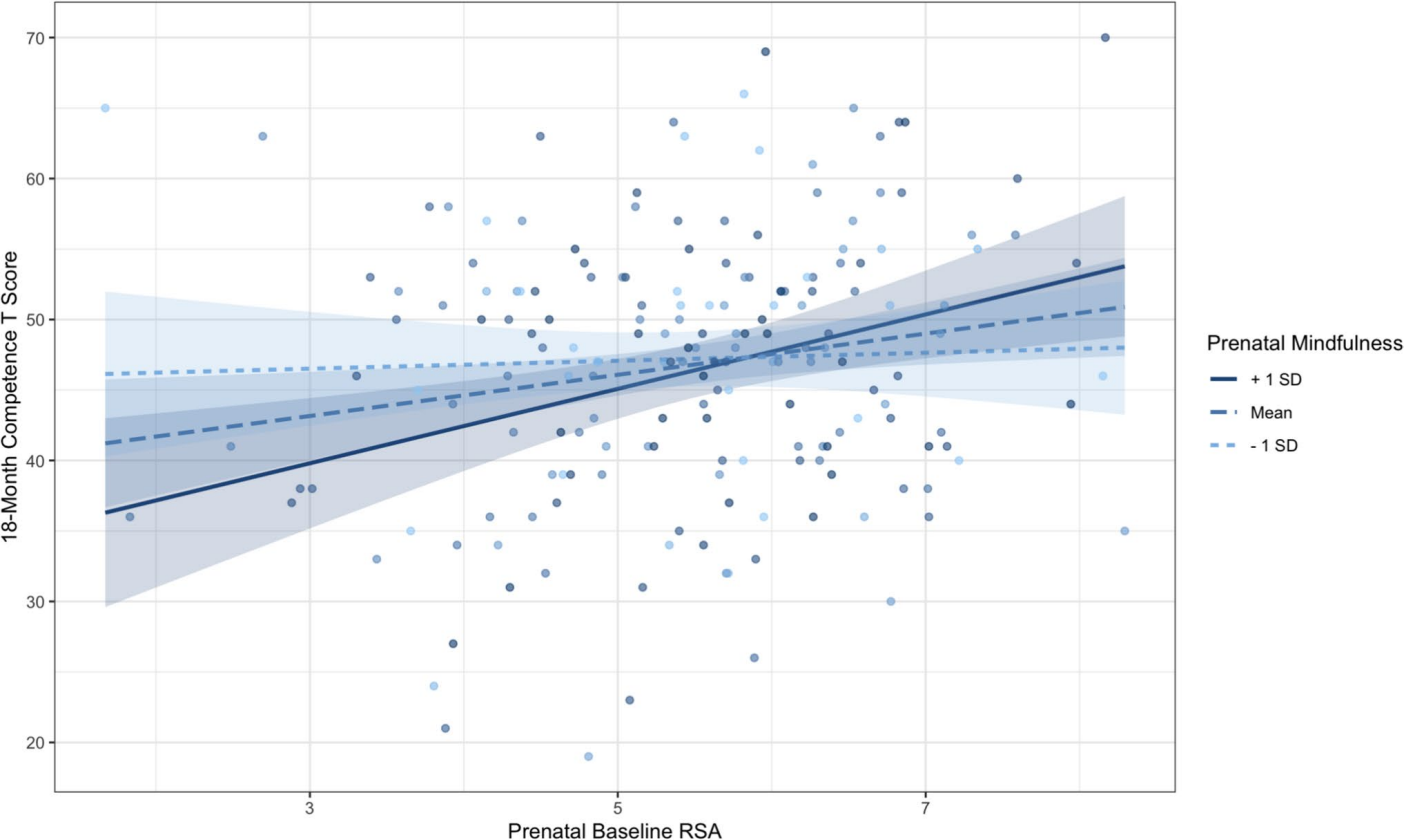
Interaction between prenatal maternal baseline RSA and prenatal maternal mindfulness predicting toddler competence at 18-months with 95% confidence intervals. The simple slopes for high prenatal mindfulness (+ 1 *SD*; solid line) and moderate prenatal mindfulness (Mean; dashed line) were significant (*p* < .01), while the slope for low mindfulness was not significant

### The Moderating role of Maternal Mindfulness

We next examined whether maternal mindfulness moderated associations between prenatal self-reported emotion dysregulation and toddlers’ ITSEA outcomes. There was a significant interaction when predicting toddler competence. Simple slopes testing revealed that higher emotion dysregulation was related to lower toddler competence only for women with higher (1 *SD* above the mean) mindfulness (β = −0.24, *p* = .04, 95% CI [−0.47, −0.01]). This effect was significant when mindfulness was outside the interval [–1.90, 0.83] when the range of observed values for mindfulness is [−2.44, 1.70]. Self-reported emotion dysregulation was not significantly associated with toddler competence at the mean (β = −0.07, *p* = .47, 95% CI [−0.25, 0.12]) or 1 *SD* below the mean of maternal mindfulness (β = 0.10, *p* = .32, 95% CI [−0.11, 0.31]). Figure 1 depicts the interaction. Maternal mindfulness and prenatal self-reported emotion dysregulation did not significantly interact when predicting toddler externalizing, internalizing, or dysregulation behaviors. See Supplemental Table 3 for full results.

We next tested whether prenatal maternal mindfulness moderated the association between prenatal maternal resting RSA and toddlers’ ITSEA outcomes. Mindfulness significantly moderated the association between resting RSA and competence (β = .16, *p* = .02, 95% CI [0.03, 0.31]). Simple slopes testing revealed that maternal resting RSA was positively associated with toddler competence for women with higher (1 *SD* above the mean; β = 0.31, *p* = .001, 95% CI [0.13, 0.49]) and average mindfulness (β = 0.18, *p* = .01, 95% CI [0.04, 0.32]); specifically, this effect was significant when mindfulness was outside the interval [−10.15, 0.15]. In contrast, resting RSA was not significantly associated with competence at low levels of mindfulness (1 *SD* below the mean; β = 0.04, *p* = .48, 95% CI [−0.14, 0.23]). Figure 2 depicts the interaction. Although mindfulness was negatively associated with externalizing behaviors (β = −0.19, *p* = .01, CI [−0.32, −0.05]) and dysregulation (β = −0.15, *p* = .02, 95% CI [−0.27, −0.03]), mindfulness and prenatal baseline RSA did not significantly interact when predicting toddler externalizing, internalizing, or dysregulation behaviors. See Supplemental Table 3 for full results.

Supplemental models substituting maternal psychopathology for emotion dysregulation were not statistically significant (see Supplemental Materials and Supplemental Table 4).

## Discussion

The current study extends our understanding of how prenatal maternal emotion dysregulation—measured via both self-report and maternal resting RSA—relates to toddler socioemotional functioning at 18 months. This dual-method approach reflects the multilevel nature of emotion dysregulation, a construct that encompasses subjective, cognitive, and behavioral components as well as physiological regulatory processes. Self-report captures mothers’ perceptions of their emotional experiences and behaviors, while resting RSA indexes physiological regulatory capacity within the parasympathetic nervous system. Our findings reflect this distinction in processes as greater self-reported emotion dysregulation was associated with higher levels of toddler internalizing, externalizing, and dysregulation behaviors, while higher maternal resting RSA was associated with greater toddler competence. These results provide novel evidence that subjective and physiological facets of prenatal emotion dysregulation may relate differentially to toddler socioemotional development. In addition, prenatal maternal baseline RSA and self-reported dysregulation were associated with social competence at *higher* levels of maternal mindfulness. Higher and moderate levels of maternal mindfulness may enhance the positive effects of physiological regulation that promotes toddler competence. However, when paired with higher self-reported emotion dysregulation, higher levels of mindfulness may heighten mothers’ awareness of their dysregulation and distress without adequate coping strategies, potentially contributing to lower toddler competence. Thus, mindfulness may amplify both adaptive and maladaptive maternal regulatory tendencies that may lead to divergent outcomes.

### Prenatal Emotion Dysregulation and Toddler Socioemotional Functioning

The first aim of this study was to examine whether self-reported emotion dysregulation and maternal resting RSA during pregnancy predicted toddler socioemotional functioning. As hypothesized, higher prenatal emotion dysregulation was linked to greater socioemotional difficulties at 18 months, including more internalizing, externalizing, and dysregulation behaviors. This is consistent with prior research associating prenatal maternal psychological distress to children’s emerging emotional and behavioral difficulties (Duguay et al., 2022; Monk et al., 2019). These findings are broadly aligned with the DOHaD framework, which suggests that maternal stress and dysregulation during pregnancy may influence fetal neurodevelopment, thereby increasing vulnerability to later psychopathology (Barker, 1998, 2007; Gluckman et al., 2016). However, it is also likely that these associations reflect both prenatal and postnatal influences. For example, maternal emotion dysregulation may persist postpartum, contributing to stress or dysregulated parenting, which could further contribute to child socioemotional difficulties. Importantly, although we did not directly control for postnatal maternal emotion dysregulation, in line with our preregistration, prior evidence indicates that prenatal anxiety remains associated with infant negative affectivity even when accounting for both prenatal and postnatal anxiety symptoms (Zhou et al., 2024), supporting the plausibility of a prenatal programming pathway. Longitudinal, mechanistic studies are needed to disentangle these prenatal effects from ongoing environmental factors.

Although self-reported emotion dysregulation did not significantly predict toddler competence, higher maternal resting RSA was associated with greater toddler competence. Importantly, resting RSA did not significantly predict toddler internalizing, externalizing, or dysregulation behaviors. These findings suggest that physiological regulatory capacity, as indexed by maternal autonomic activity during pregnancy, may be particularly relevant for supporting foundational abilities of attention, imitation, compliance, and social engagement, beyond mothers’ subjective perceptions of their own emotion dysregulation. Specifically, mothers with higher prenatal resting RSA, which may reflect greater physiological flexibility and stress resilience (Beauchaine, 2015; Porges, 2007), may be more likely to engage in responsive caregiving postpartum. Another possibility is that prenatal exposure to high resting RSA could influence the fetal parasympathetic nervous system. The mother’s vagus nerve innervates the uterus (Lychkova et al., 2014), suggesting a possible mechanism by which maternal RSA could impact development of fetal RSA. Finally, there could be a role of unassessed genetic influences. Mothers with higher resting RSA could have toddlers with higher resting RSA, which has been related to greater socioemotional competence and lower risk for behavioral problems in early childhood (Morales et al., 2015; Porges, 2007). However, resting RSA captures only one dimension of physiological regulation. Zhou et al. (2024) found that prenatal RSA reactivity, but not resting RSA, predicted toddler internalizing problems at 18 months, which was mediated by infant negative affectivity at 7 months (Zhou et al., 2024). Future studies should integrate both baseline and task-based physiological indicators to better understand how maternal regulation shapes early competence.

### The Protective Role of Maternal Mindfulness

The second aim of this study was to examine whether prenatal maternal mindfulness, or everyday mindful attention and awareness as measured in this study, moderated relations between prenatal emotion dysregulation and toddler socioemotional outcomes. Importantly, the MAAS primarily assesses everyday mindful attention and awareness and does not capture other facets of mindfulness often emphasized in intervention research, such as nonjudgemental acceptance. Findings indicated that maternal mindfulness differentially moderated effects of prenatal emotion dysregulation and maternal resting RSA on toddler competence, suggesting that mindfulness plays a nuanced role in shaping early socioemotional development.

Specifically, at higher levels of mindfulness, higher self-reported prenatal emotion dysregulation was associated with lower competence in toddlers. Although mindfulness is typically associated with improved emotion regulation, it is often examined in the context of interventions. However, this study captured everyday mindful attention and awareness to measure mindfulness in a non-intervention context. Therefore, greater mindfulness for mothers with high self-reported emotion dysregulation may simply reflect heightened attention to and awareness of distress without providing the necessary strategies for coping. This may, in turn, undermine caregiving quality or the parent’s ability to buffer stress, ultimately affecting the child’s emerging competence. Indeed, treatments targeting emotion dysregulation that only include mindfulness skills sometimes have iatrogenic effects (Simon et al., 2022), highlighting the importance of incorporating broader emotion regulation strategies.

In contrast, maternal mindfulness magnified the protective effect of resting RSA on toddler competence. This suggests that mindfulness may enhance the regulatory benefits of physiological flexibility during pregnancy, potentially by supporting adaptive caregiving responses postpartum. This finding aligns with research showing that mindfulness promotes autonomic flexibility and engagement with social and emotional cues (Schuman-Olivier et al., 2020). Taken together, these findings suggest mindfulness may not be universally protective but instead depends on maternal emotional and physiological resources.

Mindfulness does not appear operate to uniformly across maternal regulatory domains. Our findings could help explain mixed evidence on the efficacy of mindfulness-based interventions for improving autonomic functioning during pregnancy (Brown et al., 2021). They also raise concerns about possible iatrogenic effects when mindfulness increases awareness without addressing underlying distress (Valdez et al., 2016). At the same time, prior studies in perinatal populations highlight promising benefits of mindfulness for maternal and child outcomes (Lucena et al., 2020). Therefore, interventions aimed at reducing prenatal emotion dysregulation should approach mindfulness carefully, ensuring that practices are tailored to individual needs and not applied uniformly. As mindfulness-based interventions gain momentum and show potential to influence pathways of intergenerational mental health, it is essential to identify for whom and under what conditions they are most effective.

### Limitations and Future Directions

This study offers several important contributions to the growing literature on prenatal maternal dysregulation and child socioemotional development. Notably, the sample was recruited to reflect a broad spectrum of emotion dysregulation, including intentional oversampling of individuals with elevated emotion dysregulation, which strengthens the generalizability of findings across varying levels of psychological risk. This transdiagnostic indicator captures risk processes that cut across multiple forms of psychopathology and offers a more nuanced predictor than diagnostic categories alone; however, we cannot determine whether specific psychopathology categories may be driving these effects, highlighting an important direction for future work. The inclusion of both self-reported and physiological indicators of maternal emotion dysregulation allows for a multidimensional assessment of prenatal influences. Additionally, the socioeconomic diversity of the sample reflects the broader population of Salt Lake City, Utah enhancing the ecological validity of the results. By examining maternal mindfulness as a moderator, the study provides a nuanced look at how regulatory capacities interact in shaping early child outcomes—an area that remains underexplored in the developmental literature.

At the same time, several limitations should be noted. The reliance on self-report measures for maternal emotion dysregulation, mindfulness, and toddler socioemotional functioning may introduce bias. Further, the DERS and MAAS may have conceptual overlap, particularly in the DERS subscales assessing emotional awareness and clarity, which could complicate the interpretation of their unique associations with toddler outcomes. However, these subscales were not more highly correlated with the MAAS than the other DERS subscales (Supplemental Table 5), suggesting that the observed associations with toddler outcomes are not solely driven by shared variance in awareness, but rather reflect broader aspects of maternal emotion dysregulation. Additionally, the MAAS primarily assesses present-moment attention and awareness and does not capture the full multidimensional construct of mindfulness emphasized in many intervention models. Although the measures used have demonstrated strong reliability and validity, future studies would benefit from incorporating multi-method approaches, including observational assessment or intervention, ecological momentary assessment, and physiological markers of infant stress reactivity.

Additionally, because the sample was intentionally selected to include mothers with high and low levels of emotion dysregulation, findings may not generalize to the broader population and could modestly overestimate effect sizes. However, this sampling strategy increased variability and improved power to detect associations across the full spectrum of maternal emotion dysregulation. While the study provides meaningful evidence of prenatal effects on toddler outcomes, postnatal factors such as caregiving behaviors, parenting stress, and attachment likely shape toddler socioemotional trajectories. Future studies should adopt longitudinal, multi-level designs that integrate prenatal and postnatal influences. Finally, our finding that higher maternal mindfulness amplified the effects of prenatal emotion dysregulation on toddler competence raises novel questions about the conditional utility of mindfulness in high-distress contexts. It suggests that mindfulness, when paired with emotion dysregulation or lack of self-compassion, may heighten awareness of distress without bolstering coping resources. Future research should examine whether specific facets of mindfulness (e.g., acceptance vs. attentional awareness) or related constructs such as self-compassion are more predictive of adaptive outcomes.

## Conclusions

This study offers novel evidence that prenatal maternal emotion dysregulation—measured via self-report and physiological dysregulation—uniquely predicts toddler socioemotional functioning. Greater emotion dysregulation was linked to more behavioral difficulties, while higher resting RSA predicted greater competence, suggesting distinct developmental pathways. Maternal mindfulness moderated these effects, strengthening the link between RSA and competence but unexpectedly amplifying the negative impact of emotion dysregulation. These findings underscore the need to consider both subjective and physiological dysregulation in early development and suggest that individual differences in prenatal mindful attention and awareness may interact with maternal regulatory processes in shaping early socioemotional development. Given that stress and regulatory difficulties are disproportionately experienced by marginalized groups perhaps due to structural inequities, this research highlights potential pathways for reducing early-emerging disparities in mental health through targeted, equitable intervention efforts. Future work should also explore underlying mechanisms using multi-method designs and examine the potential role of postnatal influences and self-compassion–based interventions.

## Supplementary Information

Below is the link to the electronic supplementary material.

Supplementary file1 (DOCX 2092 KB)

## Data Availability

The data that support the findings of this study are available from the NIMH Data Archive (NDA): https://nda.nih.gov/edit_collection.html?id=3240. All hypotheses and analytic approaches were pre-registered and can be accessed on the Open Science Framework (https://osf.io/7rsxc/overview?view_only=df79e0ac87264d18b26375989d36cb32).
