# Supplementary material for: Prenatal Emotion Dysregulation, Respiratory Sinus Arrhythmia, and Mindfulness Predict Toddler Socioemotional Development
Source: Res Child Adolesc Psychopathol. 2026 Apr 29;54(3):68. doi: 10.1007/s10802-026-01454-x (PMC13128711; doi:10.1007/s10802-026-01454-x)
Supplement: Supplementary file 1 — Supplementary file1 (DOCX 2092 KB) [file 10802_2026_1454_MOESM1_ESM.docx]

**Supplemental Methods**

Maternal psychopathology symptoms: We used the Achenbach System of Empirically Based Assessment to estimate clinical levels of psychopathology symptoms in mothers (i.e., > 70). The Achenbach is a 120-item, self-report questionnaire that evaluates symptoms of adult psychopathology, and has acceptable test-retest reliability, internal consistency, and construct validity (Achenbach et al., 2003). Mothers reported their behavior, thoughts, and feelings over the past 6-months (e.g., “my moods or feelings change suddenly”) using a 3-point Likert scale: 0 = Not at all true, 2 = Very true or often true. We utilized the depression, anxiety, somatic symptoms, avoidant behavior, attention deficit and hyperactivity, and antisocial personality problems scales for our post hoc analyses.

**Post Hoc Analyses and Results**

As a follow-up, we replaced emotion dysregulation with a general psychological symptom variable (yes/no for reporting any symptoms in the clinical range) and found no statistically significant results (Table S4), suggesting that emotion dysregulation captures more nuanced information than broad maternal psychopathology categories; however, given limited power to test each clinical category separately, we cannot rule out independent effects, highlighting an important direction for future research.

**Supplemental Table 1.** Infant and Toddler Social and Emotional Assessment (ITSEA).

| **ITSEA Domains** | **Example** |
| --- | --- |
| **Externalizing Behavior (24 items)** |  |
| Activity/Impulsivity (6 items) | Is restless and can’t sit still |
| Aggression/Defiance (12 items) | Misbehaves to get attention from adults |
| Peer Aggression (6 items) | Teases other children |
| **Internalizing Behavior (30 items)** |  |
| Depression/Withdrawal (9 items) | Seems withdrawn |
| General Anxiety (10 items) | Seems nervous, tense, or fearful |
| Separation Distress (6 items) | Cries or hangs on to you when you try to leave |
| Inhibition to Novelty (5 items) | Is shy with new children |
| **Dysregulation (34 items)** |  |
| Negative Emotionality (13 items) | Is impatient or easily frustrated |
| Sensory Sensitivity (7 items) | Is bothered by loud noised or bright lights |
| Sleeping problems (5 items) | Has trouble falling asleep or staying asleep |
| Eating problems (9 items) | Refused food that requires chewing |
| **Competence (37 items)** |  |
| Compliance (8 items) | Tries to do as you ask |
| Attention (5 items) | Plays with toys for 5 minutes or longer |
| Imitation/Play (6 items) | Hugs or feeds dolls or stuffed animals |
| Mastery Motivation (6 items) | Enjoys challenging activities |
| Empathy (7 items) | Is worried or upset when someone is hurt |
| Prosocial Peer Relations (5 items) | Plays well with other children |
| **Additional Indices (31 items)** |  |
| Maladaptive (13 items) | Has body tic or twitch s/he seems unable to control |
| Social Relatedness (10 items) | Smiles back at you from across a room |
| Atypical (8 items) | Puts things in a special order, over and over |

**Supplemental Table 2.** Standardized estimates and 95% confidence intervals for associations between prenatal emotion dysregulation and toddler socioemotional outcomes

| Predictor | Externalizing | Internalizing | Dysregulation | Competence |
| --- | --- | --- | --- | --- |
| Model 1:  DERS | **0.20**  **[0.07, 0.34]** | **0.13**  **[0.01, 0.25]** | **0.18**  **[0.06, 0.29]** | -0.01  [-0.16, 0.13] |
| Model 2:  Maternal RSA | -0.08  [-0.21, 0.06] | -0.01  [-0.13, 0.12] | -0.09  [-0.21, 0.04] | **0.16**  **[0.02, 0.30]** |

**Supplemental Table 3.** Standardized estimates and 95% confidence intervals for associations between prenatal emotion dysregulation, prenatal maternal mindfulness, and their interaction with toddler socioemotional outcomes

| Predictor | Externalizing | Internalizing | Dysregulation | Competence |
| --- | --- | --- | --- | --- |
| Model 3: DERS  DERS  Prenatal Mindfulness  DERS x Mindfulness | 0.13  [-0.06, 0.31]  -0.13  [-0.32, 0.05]  0.05  [-0.11, 0.20] | 0.13  [-0.03, 0.28]  -0.02  [-0.19, 0.14]  0.04  [-0.09, 0.16] | 0.14  [-0.02, 0.29]  -0.09  [-0.25, 0.07]  -0.10  [-0.03, 0.23] | -0.08  [-0.27, 0.12]  -0.06  [-0.25, 0.14]  **-0.19**  **[-0.35, -0.03]** |
| Model 4: Maternal RSA  RSA  Prenatal Mindfulness  RSA x Mindfulness | -0.06  [-0.19, 0.07]  **-0.19**  **[-0.32, -0.05]**  -0.07  [-0.20, 0.07] | -0.02  [-0.14, 0.11]  -0.09  [-0.22, 0.03]  -0.07  [-0.20, 0.05] | -0.09  [-0.22, 0.03]  **-0.15**  **[ -0.27, -0.03]**  -0.04  [-0.17, 0.08] | **0.17**  **[0.03, 0.31]**  -0.04  [-0.18, 0.10]  **0.17**  **[0.03, 0.31]** |

**Supplemental Table 4.** Standardized estimates and 95% confidence intervals for associations between prenatal maternal psychopathology, prenatal maternal mindfulness, and their interaction with toddler socioemotional outcomes

| Predictor | Externalizing | Internalizing | Dysregulation | Competence |
| --- | --- | --- | --- | --- |
| Model 5: Psychopathology  Prenatal Mindfulness  Psychopathology x Mindfulness | –0.02  [-0.20, 0.17]  -0.27  [-0.46, 0.08]  0.04  [-0.18, 0.25] | 0.08  [-0.09, 0.24]  -0.07  [-0.26, 0.12]  -0.02  [-0.21, 0.17] | 0.12  [-0.05, 0.28]  -0.18  [-0.37, 0.00]  0.10  [-0.09, 0.29] | -0.08  [-0.28, 0.11]  -0.02  [-0.23, 0.19]  -0.09  [-0.32, 0.14] |

**Supplemental Table 5.** Correlation of DERS subscales with MAAS

| Variable | *M* | *SD* | 1 | 2 | 3 | 4 | 5 | 6 |
| --- | --- | --- | --- | --- | --- | --- | --- | --- |
| 1. Prenatal maternal mindfulness | 4.24 | 0.86 |  |  |  |  |  |  |
| 2. Lack of emotional awareness | 13.26 | 4.76 | -.41** |  |  |  |  |  |
|  |  |  | [-.49, -.32] |  |  |  |  |  |
| 3. Lack of emotional clarity | 9.98 | 3.50 | -.50** | .66** |  |  |  |  |
|  |  |  | [-.57, -.42] | [.61, .72] |  |  |  |  |
| 4. Nonacceptance of emotional responses | 14.22 | 6.17 | -.51** | .41** | .59** |  |  |  |
|  |  |  | [-.58, -.43] | [.32, .49] | [.52, .65] |  |  |  |
| 5. Difficulty in goal-directed behavior | 13.70 | 4.89 | -.46** | .27** | .50** | .59** |  |  |
|  |  |  | [-.54, -.38] | [.18, .36] | [.42, .57] | [.52, .65] |  |  |
| 6. Impulse control difficulties | 11.55 | 4.94 | -.42** | .38** | .57** | .60** | .63** |  |
|  |  |  | [-.50, -.33] | [.29, .47] | [.50, .63] | [.53, .66] | [.56, .69] |  |
| 7. Limited emotion regulation strategies | 17.37 | 6.99 | -.55** | .45** | .64** | .74** | .74** | .76** |
|  |  |  | [-.62, -.48] | [.36, .53] | [.57, .69] | [.69, .78] | [.70, .79] | [.71, .80] |


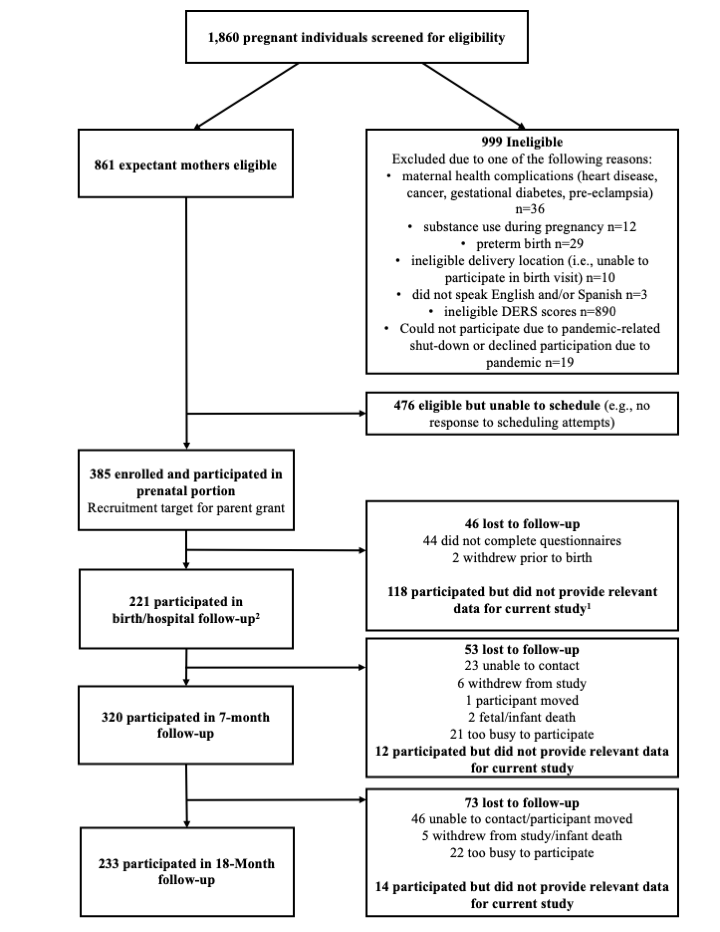


**Supplemental Figure 1**. STROBE for participant recruitment.


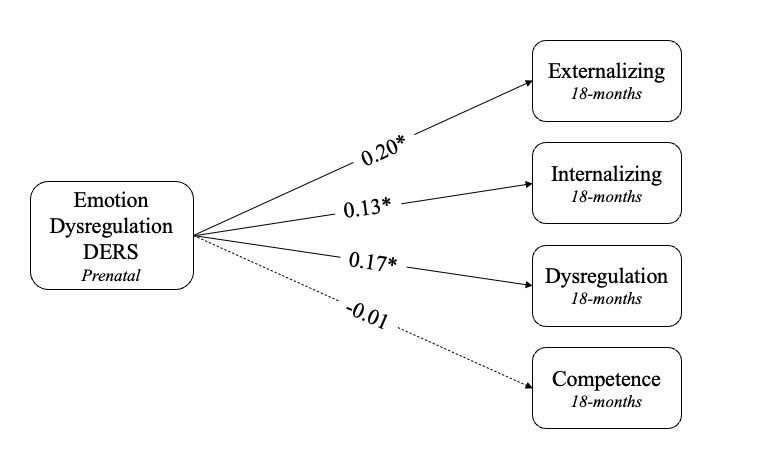


**Supplemental Figure 2.** Path model 1 examining associations with prenatal emotion dysregulation (DERS) and toddler socioemotional outcomes (ITSEA). Significant associations are indicated by solid lines, and non-significant associations are dashed.


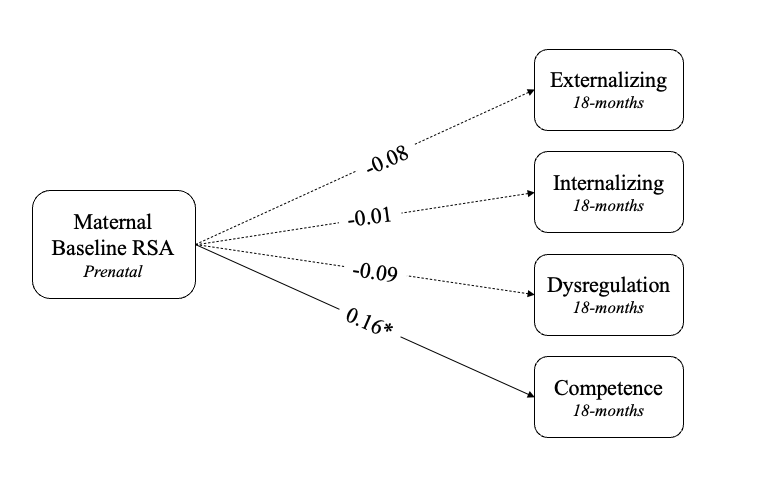


**Supplemental Figure 3.** Path model 2 examining associations with prenatal emotion dysregulation (DERS) and toddler socioemotional outcomes (ITSEA). Significant associations are indicated by solid lines, and non-significant associations are dashed.
